# More than a bystander: the contributions of intrinsic skeletal muscle defects in motor neuron diseases

**DOI:** 10.3389/fphys.2013.00356

**Published:** 2013-12-18

**Authors:** Justin G. Boyer, Andrew Ferrier, Rashmi Kothary

**Affiliations:** ^1^Ottawa Hospital Research Institute, Regenerative Medicine ProgramOttawa ON, Canada; ^2^Department of Cellular and Molecular Medicine, University of OttawaOttawa, ON, Canada; ^3^Department of Medicine, University of OttawaOttawa, ON, Canada

**Keywords:** mouse models, neuromuscular disease, myofiber degeneration, fusion defect, insulin-like growth factor 1

## Abstract

Spinal muscular atrophy (SMA), amyotrophic lateral sclerosis (ALS), and spinal-bulbar muscular atrophy (SBMA) are devastating diseases characterized by the degeneration of motor neurons. Although the molecular causes underlying these diseases differ, recent findings have highlighted the contribution of intrinsic skeletal muscle defects in motor neuron diseases. The use of cell culture and animal models has led to the important finding that muscle defects occur prior to and independently of motor neuron degeneration in motor neuron diseases. In SMA for instance, the muscle specific requirements of the SMA disease-causing gene have been demonstrated by a series of genetic rescue experiments in SMA models. Conditional ALS mouse models expressing a muscle specific mutant *SOD1* gene develop atrophy and muscle degeneration in the absence of motor neuron pathology. Treating SBMA mice by over-expressing IGF-1 in a skeletal muscle-specific manner attenuates disease severity and improves motor neuron pathology. In the present review, we provide an in depth description of muscle intrinsic defects, and discuss how they impact muscle function in these diseases. Furthermore, we discuss muscle-specific therapeutic strategies used to treat animal models of SMA, ALS, and SBMA. The study of intrinsic skeletal muscle defects is crucial for the understanding of the pathophysiology of these diseases and will open new therapeutic options for the treatment of motor neuron diseases.

## Introduction

Everything from physical exercise to daily chores and even breathing depends on force generated by skeletal muscles. For a skeletal muscle to produce a contraction, a signal in the form of an action potential is required. The motor neuron is responsible for providing this required signal. The site where the motor neuron joins the muscle is called the neuromuscular junction and together, the motor neuron and the muscle are referred to as the motor unit.

Defects in the motor unit can seriously impact muscle contraction generation and lead to severely disabling diseases. Motor neuron diseases consist of a group of conditions characterized by motor neuron loss and atrophy of the associated musculature. Spinal muscular atrophy (SMA), amyotrophic lateral sclerosis (ALS), and spinal-bulbar muscular atrophy (SBMA) are examples of such motor neuron diseases. Although these diseases have quite different etiologies, SMA, ALS, or SBMA are all typified by progressive paralysis resulting in severe disability, and are commonly fatal.

Although the importance of motor neuron pathology is well-established in these diseases, recent work has revealed an involvement of other cell types, including myocytes, in the pathogenic process (Bricceno et al., 2012a; Hamilton and Gillingwater, 2013). Muscle weakness and atrophy are often tightly associated with motor neuron pathology. Importantly, however, use of both cell culture and conditional mouse models has revealed defects in skeletal muscle that occur in the absence of defective motor neurons. Such studies highlight a potential contribution of skeletal muscle defects to the symptoms of SMA, ALS, and SBMA patients. These findings have major implications for the development of therapeutics for these diseases. In the current review, we discuss the latest findings regarding intrinsic muscle defects as well as muscle-specific therapeutic strategies to treat SMA, ALS, and SBMA.

## Spinal muscular atrophy

### Disease characteristics

SMA represents the leading genetic cause of infant death, affecting 1 in 10,000 live births per year (Pearn, 1978; Prior et al., 2010). Clinically, it is typified by progressive muscle weakness and loss of alpha motor neurons from the spinal cord. SMA is caused by mutations or deletions in the *SMN1* gene (Lefebvre et al., 1995). Due to a second, partially functionally copy, named *SMN2*, SMA is a disease of low SMN levels, rather than no SMN (Brzustowicz et al., 1993; Rochette et al., 2001). The clinical severity of SMA is categorized into 4 main types, which vary in their time of onset and expected prognosis (reviewed in Boyer et al., 2010). Furthermore, *SMN2* serves as a disease modifier since the copy number of the *SMN2* gene in SMA patients modulates disease severity.

The SMN protein is ubiquitously expressed and localizes to the nucleus and cytoplasm (Liu and Dreyfuss, 1996). In the nucleus, SMN is present in gemini of coiled bodies (gems) which are structures associated with Cajal bodies (Liu and Dreyfuss, 1996; Coovert et al., 1997; Lefebvre et al., 1997). Here, SMN forms a complex with Gemin 2–8 as well as with Sm proteins to regulate small nuclear ribonucleoprotein (snRNP) biogenesis (Liu and Dreyfuss, 1996; Meister et al., 2000; Pellizzoni et al., 2002; Ogawa et al., 2007). SnRNPs are essential for pre-mRNA splicing and a decrease in SMN produces a reduction in snRNP assembly in SMA patients (Will and Luhrmann, 2001; Wan et al., 2005). In additional to SMN's established role in snRNP biogenesis, it has also been suggested to have a role in transcription and mRNA transport (Pellizzoni et al., 2001; Meister et al., 2002; Paushkin et al., 2002; Rossoll et al., 2003; Kariya et al., 2008).

Since the identification of SMN as the disease-causing gene in SMA, a number of mouse models have been generated to gain insight into the pathogenic process. *Smn* knockout mice (*Smn*^−/−^) are embryonic lethal while heterozygous (*Smn*^+/−^) mice can be used as a model of very mild SMA (Schrank et al., 1997). Mice do not harbor the *SMN2* gene as it is unique to humans, but by introducing low copies of the human *SMN2* gene into the *Smn*^−/−^background, researchers were able generate a mouse model (*Smn*^−/−^*;SMN2*) mimicking the severe SMA phenotype (Monani et al., 2000). Although the *Smn*^−/−^*;SMN2* model is genetically representative of human patients, these mutant mice are very small in size and have a short life span making them difficult models to work with, especially when assessing therapeutics. The recent creation of milder SMA model mice has led to the discovery that cell types other than motor neurons are also affected in SMA (Hammond et al., 2010; Michaud et al., 2010; Bowerman et al., 2012a,b; Osborne et al., 2012; Cobb et al., 2013). These mice will also allow for the identification of new mechanisms of disease and will facilitate testing of therapeutic approaches.

### Muscle intrinsic defects

As motor neurons and skeletal muscle are both functionally and structurally connected, it is difficult to study skeletal muscle defects independently from diseased motor neurons. The use of cell culture and conditional mouse models has, however, allowed researchers to study the role of Smn in skeletal muscle. Shafey et al. (2005) generated a series of hypomorphic cell lines in which Smn expression was depleted to varying levels in C2C12 myoblasts. Upon reducing Smn levels, defects such as decreased proliferation, aberrant myoblast fusion and malformed myotubes became apparent. These findings demonstrate that low Smn levels can result in intrinsic muscle defects and suggest a role for Smn in myoblast fusion. *In vivo*, the importance of Smn has been highlighted by conditional knockout mice in which the muscle-specific knockout of *Smn* was achieved using a floxed Smn allele with Cre recombinase controlled by the *human skeletal actin* (*HSA*) muscle-specific promoter (Cifuentes-Diaz et al., 2001). These mice develop a phenotype reminiscent of that observed in mouse models of muscular dystrophy. Skeletal muscle fibers from these mice have a disorganized sarcolemma and the mice have a reduced lifespan without displaying any overt neuropathology (Cifuentes-Diaz et al., 2001; Nicole et al., 2003). Although the results from conditional mouse studies have demonstrated a need for Smn in skeletal muscle, advances in animal genetic manipulation now allows for conditional deletion of Smn with residual *SMN2* expression. Therefore, we propose that skeletal muscle conditional SMA model mice be revisited using genetics reminiscent of the human disease.

Studies demonstrating muscle intrinsic defects have also been performed using human primary myoblasts from SMA patients. Upon differentiation of human myoblasts, myotubes from SMA patients displayed a reduction in fusion (Arnold et al., 2004), a result reminiscent of what was observed in the C2C12 Smn-knockdown cells. Furthermore, skeletal muscle defects such as vacuolization and compromised sarcomere structures were reported in human SMA muscle cells co-cultured with rat spinal cord explants (Braun et al., 1995). Although these primary cell culture studies were fairly concise, they nonetheless provide valuable insight into intrinsic muscle abnormalities. Collectively, the cell culture and conditional mouse studies were the first to show muscle intrinsic defects in SMA, and establish and validate a strong rationale to study muscle defects in the context of SMA.

### Abnormal skeletal muscle development in SMA mice

A growing body of evidence suggests that Smn-depletion leads to aberrant skeletal muscle development in SMA model mice. In the *Smn*^−/−^;*SMN*2;Δ7 mouse model, Lee et al. (2011) showed that the cross-sectional area of myofibers did not increase from P5 to P13 suggesting impaired muscle growth. Similarly, the tibialis anterior muscle from phenotype stage *Smn*^−/−^;*SMN*2 mice show similar cross-sectional area as that in pre-symptomatic mice (Dachs et al., 2011). A study on patient samples confirms that SMA muscles demonstrate impaired growth and maturation characterized by smaller and disorganized myotubes (Martinez-Hernandez et al., 2009). Collectively, these studies point to defects in muscle growth, however, the molecular basis for this is still unclear. One possibility for the reduced myofiber size in muscles from SMA model mice is that it is due to a defect in post-natal muscle development. Delayed muscle development is supported by the increased expression of immature myosin heavy chain (MHC) isoform in SMA model mice. Sustained expression of the embryonic and perinatal MHC isoforms has been detected at the transcript and protein level in phenotype stage *Smn*^−/−^;*SMN*2;Δ7 mice (Kong et al., 2009; Lee et al., 2011). Furthermore, reduced expression of fast MHC isoforms has been reported in skeletal muscles of SMA model mice (Biondi et al., 2008; Lee et al., 2011). The transition between developmental MHC isoforms and fast MHC isoforms occurs later in development compared to slow MHC isoforms (Schiaffino and Reggiani, 2011), therefore delayed muscle development could account for the decreased expression of type 2B and 2× fibers in SMA muscles. The aberrant MHC expression profile was also associated with a delay in postsynaptic endplate development. The expression of the immature acetylcholine receptor persists in severe SMA model mice and is accompanied by impaired postsynaptic maturation at the morphological level (Kariya et al., 2008; Kong et al., 2009; Dachs et al., 2011; Bowerman et al., 2012a). A recent study assessed the functional capacities of skeletal muscle and demonstrated muscle weakness was an early feature of pathology in multiple mouse models of SMA (Boyer et al., 2013). Moreover, the severe muscle weakness was associated with delayed expression of mature isoforms of muscle function proteins such as ryanodine receptors and sodium channels. For the moment, direct evidence demonstrating that muscle weakness in SMA model mice is due to the delayed myogenesis and aberrant expression of muscle function proteins is lacking. Nonetheless, together these results suggest a role for Smn in skeletal muscle development. Further, it suggests that low levels of Smn lead to impaired muscle maturation, and thereby causing muscle weakness.

Hayhurst et al. (2012) have provided evidence suggesting that Smn-depletion leads to accelerated muscle differentiation *in vivo*. Satellite cells, identified by the paired-box transcription factor Pax7, were increased in number and expressed myogenic markers earlier in *Smn*^−/−^;*SMN*2 mice compared to controls. Despite the accelerated expression of the myogenic regulatory factors in *Smn*^−/−^;*SMN*2 satellite cells, these cells failed to grow upon differentiation in culture (Hayhurst et al., 2012).

How low levels of Smn lead to specific developmental defects in SMA model mice is unclear at the moment. However, several possible scenarios can be envisaged. First, Smn has specific function(s) during development through its association with one or more binding partners or second that low levels of Smn disrupts a major cellular process such as splicing which could impact on the expression of multiple genes important for muscle development and function. Thirdly, as muscle development is orchestrated by activity, defects at the levels of the motor neuron may negatively impact upon the expression of factors controlling post-natal muscle maturation.

### Targeting pathways of muscle atrophy in SMA

Although several attempts have been made to promote muscle growth in SMA model mice, few studies have begun addressing the underlying mechanisms leading to muscle atrophy in the disease. During skeletal muscle atrophy caused by denervation, myogenin is up-regulated and directly stimulates the expression of muscle-specific ubiquitin E3 ligases, namely atrogin 1 and muscle RING-finger protein 1 (MuRF1), collectively referred to as atrogenes (Moresi et al., 2010). Substrates for atrogin 1 include MyoD, and eIF3f, which is an activator of protein synthesis, while sarcomeric proteins such as myosins and troponins are substrates of MuRF1 (Bonaldo and Sandri, 2013). Deletion of either atrogin 1 or MuRF1 leads to complete protection from denervation-induced muscle atrophy (Bodine et al., 2001).

Recently, a study by Bricceno et al. (2012b) demonstrated that following phenotype onset, a robust increase in atrogene expression was observed in SMA model mice (Table 1). Increased myogenin expression correlated with the up-regulation of the atrogenes, suggesting that the atrophy was mediated by muscle denervation. The expression of myogenin and atrogenes was also increased in skeletal muscle samples from human patients. Administration of the histone deacetylase inhibitor trichostatin A (TSA) prior to muscle atrophy onset attenuated atrogene expression in a mouse model of SMA (Bricceno et al., 2012b). TSA treatment decreased atrogene expression in denervated mice in which atrophy is mediated by myogenin but had no effect in a starved model of atrophy. Thus, this result suggests that TSA directly impacts on the myogenin dependent atrogene pathway. Direct evidence supporting denervation of the muscles used in the study by Bricceno et al. (2012b) was not presented. Therefore, it is not fully understood whether the up-regulation of myogenin in SMA model mice is attributable to impaired myogenesis, muscle denervation or both. Currently, understanding mechanisms of atrophy in SMA is an understudied area of research. For instance, whether the autophagic pathway or calpain-mediated muscle breakdown contribute to muscle atrophy in SMA is not known but could be of therapeutic importance. Further dissection of the molecular pathways responsible for the muscle atrophy, and testing therapeutics to treat atrophy in SMA is definitely warranted.

**Table 1:** **Summary of pathways leading to muscle atrophy in motor neuron diseases**.


SMA	-	increased expression of myogenin, MuRF1 and atrogin 1 in SMA model mice and SMA human muscle samples (Bricceno et al., 2012b)
	-	TSA administration reduced the levels of atrogenes in SMA model mice (Bricceno et al., 2012b)
ALS	-	increased levels of atrogin 1 but not MuRF1 in ALS *SOD1*^*G*93*A*^ model mice and ALS human samples (Léger et al., 2006)
	-	decreased levels of AKT in ALS human samples (Léger et al., 2006)
	-	increased levels of FoxO3 and MuRF1 but not atrogin1 in *MLC/SOD1*^*G*93*A*^ animals (Dobrowolny et al., 2005)
	-	increased levels of expression of autophagy genes in *MLC/SOD1*^*G*93*A*^ mice (Dobrowolny et al., 2005)

### A role for SMN in muscle

By isolating single myofibrils from myofibers, Walker et al. (2008) were able to identify Smn as a sarcomeric protein in striated muscle from mice. Specifically, Smn localizes to the Z-disc where it interacts with the actin crosslinking protein α-actinin (Rajendra et al., 2007; Walker et al., 2008; Shafey et al., 2010). Interestingly, other members of the SMN complex such as Gemins 2, 3, 4, and 6 are also present at Z-discs. However, other proteins essential for snRNP assembly are absent, suggesting that Smn plays a novel role at this adhesion site.

Currently, it is unclear whether there are any overt cytoskeletal structural defects in muscles from SMA model mice. Since mature muscles require less Smn expression than developing muscle (La Bella et al., 1998), the residual full-length protein produced by *SMN2* may be sufficient to fulfill its role at the Z-disc. However, given the localization of Smn in skeletal muscle, the complete absence of full-length Smn protein in skeletal muscle may lead to cytoskeletal defects reminiscent of what was observed in Smn muscle-specific depleted mice. Given the domains present in the Smn protein, it is unlikely that Smn plays a structural role in mature myofibers. However, a role for Smn in mechanosensing may be possible.

### Increased cell death in SMA muscle

To gain insight into intrinsic molecular changes in skeletal muscle prior to motor neuron degeneration, Mutsaers et al. (2011) performed a proteomic screen using the rostral band of the levator auris longus muscle in pre-symptomatic severe SMA model mice. Several proteins involved in cell death pathways were aberrantly expressed, some of which were validated in skeletal muscle from human patients. Furthermore, the authors highlighted changes in protein expression that were not identified in muscle samples from denervated mice, suggesting these changes are unlikely to be due to defective muscle innervation. In a separate study, increased apoptotic cell death was observed in muscles from phenotype stage *Smn^−/−^;SMN2* mice but was not detected at the pre-symptomatic P0-P1 time point. To demonstrate that the presence of apoptotic cell death was a muscle intrinsic phenomenon, Dachs et al. (2011) performed sciatic nerve denervation in neonatal mice. Forty-eight hours post-denervation, no increase in apoptotic cells was observed in the experimentally denervated muscles compared to sham controls. Therefore, the increased cell death correlates with disease progression in *Smn*^−/−^*;SMN2* mice and is not attributed to acute muscle denervation *per se*. That said, an investigation of cell death in Smn-depleted C2C12 cells and cultured satellite cells isolated from *Smn*^−/−^*;SMN2* mice revealed normal proportion of apoptotic cells (Shafey et al., 2005; Hayhurst et al., 2012). Furthermore, the number of apoptotic cells in type 1 human SMA muscle samples was comparable to controls (Martinez-Hernandez et al., 2009). Therefore, it would appear that increased cell death in skeletal muscle might be limited to mouse models of SMA. How the absence of Smn leads to increased apoptosis and whether apoptosis in skeletal muscle is primary or secondary to the SMA pathogenesis is unclear and requires further study.

### The therapeutic requirements of SMN in skeletal muscle

Several therapeutic approaches currently being developed for the treatment of SMA involve increasing SMN levels. In an attempt to demonstrate the therapeutic requirements of SMN in neuronal and muscle tissue, a series of genetic rescues in mouse models were performed. In an initial study, the expression of Smn was driven by either the muscle-specific promoter *HSA* or by the neuron-specific *prion protein* promoter (*PrP*) (Gavrilina et al., 2008). Crossing *HSA* and *PrP* rescue mice onto the *Smn*^−/−^*;SMN2* background allowed for a direct comparison between both the neuronal and muscle approaches on survival. The longest surviving *PrP* rescued line lived an average of 210 days while the best surviving *HSA* rescued line lived an average of 160 days. Thus, these results demonstrate that rescue in either neurons or muscle can offer significant improvements in survival. However, the results from this study are challenging to interpret given that both the longest surviving muscle and neuronal rescue lines demonstrated leaky Smn protein expression in the spinal cord and in muscle, respectively. Moreover, the use of the *HSA* promoter to express Smn in skeletal muscle may not be early enough in development, and may therefore not be representative of when Smn is needed in skeletal muscle. In addition, the use of the *PrP* promoter makes it difficult to conclude that Smn is required precisely in the spinal cord since this promoter targets multiple cell types in the nervous system.

More recently, a similar study was performed using conditional SMA model mice expressing tissue-specific Cre drivers, including the motor neuron-specific choline acetyltransferase promoter (*ChAT^cre^*), and the muscle-specific myogenic determination 1 (*MyoD^cre^*) and myogenic factor 5 (*Myf5^cre^*). MyoD and Myf5 are myogenic regulatory factors expressed very early during myogenesis and it was therefore anticipated that they would yield more significant improvements in the survival of SMA mice (Martinez et al., 2012). Interestingly, restoring Smn expression in motor neurons of *ChAT^cre^* mice led to a very modest increase in survival (from 15 to 23 days) compared to the survival reported using the *PrP* promoter. The *ChAT^cre^* extended survival 2 days more than the muscle-specific promoters *MyoD^cre^* and *Myf5^cre^*. *ChAT^cre^* conditional mice showed increased SMN expression in motor neurons and improved neuromuscular junction function and morphology. Despite these improvements, only the *MyoD^cre^* and the *Myf5^cre^* lines produced a robust increase in the cross-sectional area of myofibers. It should be noted that Smn expression from the *Myf5^cre^* driver was detectable in the neuronal tissue making it somewhat difficult to interpret the results from this particular conditional mouse model (Martinez et al., 2012). Nonetheless, the results from *MyoD^cre^* and *Myf5^cre^* demonstrate the importance of restoring Smn expression in skeletal muscle and suggest that a combinatorial expression of Smn in both myofibers and motor neurons may provide a more impressive extension of life in mouse models of SMA. These data, suggest that restoration of Smn expression in skeletal muscle should not be overlooked when developing therapeutic strategies.

### Inducing muscle growth pathways in SMA model mice

In an effort to reverse the muscle atrophy phenotype in mouse models of SMA, researchers have attempted to stimulate muscle growth pathways by various means. Myostatin is a potent negative regulator of muscle mass and the modulation of the myostatin pathway leads to dramatic muscle growth (Lee and McPherron, 2001). It was therefore hypothesized that modulation of the myostatin pathway may lead to phenotypic improvements in mouse models of SMA. Interestingly, myostatin and follistatin, a myostatin antagonist, were both found to be increased in mouse models of SMA suggesting a possible compensatory attempt by the animal to minimize muscle size loss (Sumner et al., 2009). Genetic overexpression of myostatin in SMA model mice proved to have a modest effect on muscle weight but offered no improvements in motor function and survival (Rindt et al., 2012). In a similar study, the genetic overexpression of follistatin in SMA model mice did not increase myofiber size or improve lifespan (Sumner et al., 2009). However, administration of recombinant follistatin in the same SMA mouse models used as in the genetic overexpression studies had a positive effect on the disease phenotype and lifespan (Rose et al., 2009). Specifically, delivery of the recombinant follistatin increased muscle mass, body weight, improved motor function and also increased motor neuron numbers and size in SMA model mice. Collectively, these changes led to an increase in median survival time. The differences in results between both follistatin studies are unclear but are likely attributed to the different methods used to overexpress follistatin that is, genetic overexpression versus administration of recombinant protein.

Insulin-like growth factor 1 (IGF-1) is a robust positive regulator of muscle mass and the overexpression of IGF-1 in mice causes a dramatic increase in musculature (Musaro et al., 2001). Interestingly, circulating IGF-1 levels are reduced in multiple mouse models of SMA (Hua et al., 2011; Murdocca et al., 2012). Delivery of IGF-1 to the central nervous system of type III SMA model mice using an adeno-associated virus vector proved beneficial for motor neuron health but had little effect on myofiber integrity and motor function (Tsai et al., 2012). The systemic administration of IPLEX (recombinant human IGF-1 complexed with recombinant human IGF-1 binding protein 3) by intraperitoneal injection in *Smn*^−/−^*;SMN*2*;*Δ*7* animals led to increased myofiber size, reduced motor neuron cell loss but did not impact on life span and body weight of SMA model mice (Murdocca et al., 2012). Finally, combining SMN trans-splicing with an IGF-1 vector increased life span and body mass in a severe mouse model of SMA (Shababi et al., 2011). It is difficult to partial out whether the improvements in myofiber size were due to the IGF-1 protein acting on myofibers directly or rather, increased muscle size was a secondary consequence from the healthier motor neurons. The genetic muscle overexpression of IGF-1 onto the same SMA background used in the follistatin studies led to increased muscle mass as well as an increase in the median lifespan (Bosch-Marce et al., 2011). Overexpression of muscle IGF-1 had little effect on markers of muscle maturity. Therefore, IGF-1 cannot restore proper muscle development in SMA model mice. IGF-1 may prove to be of greater benefit to diseases in which myofibers have formed and matured properly and are subsequently affected by intrinsic and extrinsic pathology. In fact, promoting muscle growth using IGF-1 overexpression before the muscle has fully matured may be detrimental to the tissue. Furthermore, it should be noted that the SMA model mice were crossed to IGF-1 overexpressing heterozygotes. Therefore, the benefits of IGF-1 may have been more obvious had the mice been bred to homozygosity.

Perhaps targeting molecules more downstream where myostatin and IGF-1 pathways converge might yield more robust results. For example, Akt is downstream of both myostatin and IGF-1, and overexpression of Akt in mice has led to increased muscle size (Lai et al., 2004; Schiaffino and Mammucari, 2011). In addition to promoting muscle growth, Akt also plays an important role in inhibiting mechanisms of atrophy including inhibiting atrogenes and the autophagic pathway (Schiaffino and Mammucari, 2011).

## Amyotrophic lateral sclerosis (ALS)

### Disease characteristics

ALS is a progressive adult-onset fatal disease characterized by the selective degeneration of upper motor neurons in the cerebral cortex and lower motor neurons of the brain stem and spinal cord (Rowland and Shneider, 2001). Features of the disease include muscle weakness and atrophy, spasticity and paralysis. It has an incidence of two per 100,000 people per year in the United States (Rowland and Shneider, 2001).

The term “amyotrophic” refers to the muscle atrophy, weakness and fasciculation that signify disease of lower motor neurons, while “lateral sclerosis” refers to the hardening of lateral columns, where gliosis follows the degeneration of corticospinal tracts (Rowland and Shneider, 2001). The mean duration of survival for ALS patients is 3–5 years following disease onset, with denervation of the respiratory muscles and diaphragm generally representing the fatal event of the disease. Despite intense research efforts, limited therapeutic options remain in attenuating disease progression; nevertheless advances are being made in palliative therapy (Carter et al., 2012).

A vast majority of ALS cases are sporadic and have no known genetic component, except for missense mutations in the TAR-DNA binding protein (Kabashi et al., 2008). Inherited forms of ALS (fALS) have an autosomal dominant or recessive pattern of inheritance and constitute ~10% or less of the remaining ALS cases (Rowland and Shneider, 2001). In 1993, a breakthrough in ALS research was made with the discovery of missense mutations in the *Cu/Zn superoxide dismutase 1 (SOD1)* gene of a subset of fALS cases (Rosen et al., 1993). It is estimated that 15–20% of fALS patients harbor missense mutations in the *SOD1* gene (equating to 2% of all ALS cases).

SOD1 is a ubiquitously expressed cytosolic metalloprotease that non-covalently binds Cu and Zn. The enzyme functions by detoxifying and maintaining intracellular superoxide anions (O_2_^−^) by catalyzing the dismutation of O_2_^−^ to molecular oxygen and hydrogen peroxide. As such, mutations in SOD1 impart oxidative stress, which has become a key mechanism underlying disease pathogenesis (Barber et al., 2006). That said, to date, a number of different pathogenic mechanisms are believed to trigger ALS pathogenesis (Ilieva et al., 2009).

Most of our current knowledge of ALS pathogenic mechanisms comes from transgenic mice expressing various forms of mutant SOD1. Studies with these mice have highlighted multiple targets of damage in disease including mitochondria, proteasomes, and secretory pathways. Furthermore, while expression of mutant SOD1 within motor neurons is a primary determinant of disease onset and of an early phase of disease progression, the expression of mutant SOD1 also affects structural, physiological, and metabolic parameters in other cell types (e.g., glia and skeletal muscle) (see reviews Boillee et al., 2006; Ilieva et al., 2009). ALS is now considered a multisystemic disease, wherein a variety of cell types act synergistically to exacerbate disease pathogenesis (Ilieva et al., 2009).

The progressive paralysis in ALS is the result of degeneration and demise of motor neurons (Rowland and Shneider, 2001). Data from multiple studies suggest that toxicity is non-cell-autonomous, meaning toxicity to motor neurons derives from damage developed within cell types beyond the motor neuron (see reviews Fuchs et al., 1994; Boillee et al., 2006; Ilieva et al., 2009). Studies supporting this notion showed that ubiquitous transgenic overexpression of SOD1 mutations causing fALS leads to an ALS phenotype in mice (Wong et al., 1995), however, restricted expression of mutant SOD1 in neurons alone is not sufficient to cause this phenotype. It is perhaps important to note, however, that levels of mutant SOD1 selectively expressed in motor neurons may have been too low to initiate disease (Pramatarova et al., 2001; Lino et al., 2002). More recent studies suggest that neuron-specific expression of human mutant SOD1 in mice triggers motor neuron degeneration (Jaarsma et al., 2008; Wang et al., 2008). These discrepant studies indicate that mutant SOD1-induced motor neuron degeneration is at least partly cell autonomous, and non-neuronal mutant SOD1 expression is also likely required for disease manifestation.

### Muscle defects in ALS

Numerous studies support the notion that multiple tissues outside the CNS, including skeletal muscle (Wiedemann et al., 1998; Krasnianski et al., 2005; Dupuis et al., 2006), fibroblasts (Aguirre et al., 1998; McEachern et al., 2000), and lymphocytes (Cova et al., 2006) are affected in human ALS. In both sporadic and fALS, functional aberrations and skeletal muscle pathology are present including neurogenic-induced muscle pathology and mitochondria dysfunction (Vielhaber et al., 1999; Krasnianski et al., 2005; Echaniz-Laguna et al., 2006; Corti et al., 2009). Similarly, transgenic mice expressing mutant SOD1 recapitulate functional and metabolic deficits in skeletal muscle as seen in human ALS patients (Derave et al., 2003; Dupuis et al., 2004; Mahoney et al., 2006).

Selective expression of mutant SOD1 in mouse skeletal muscle using the myosin light chain (MLC) promoter (*MCL/SOD1*^*G*93*A*^) induced ALS-like muscle pathologies, including progressive muscle atrophy, reduced muscle strength, impaired contractility, and mitochondrial dysfunction (Dobrowolny et al., 2008). Interestingly, exclusive expression of mutant SOD1 in skeletal muscle did not trigger the degeneration of motor neurons. This finding contrasts with a similar study from Wong and Martin (2010), where human mutated SOD1 expression driven from the *HSA* promoter, resulted in pathologic phenotypes in both muscle and motor neurons reminiscent of ALS (Wong and Martin, 2010). The reasons for this discrepancy is unclear although it may be due to the fact the animals used were of a different age, being significantly younger in the work from Dobrowolny et al. (2008). Regardless of this, together these studies provide strong evidence that mutant SOD1 is toxic to skeletal muscle and challenged the accepted dogma that motor neuron degeneration, caused by the overexpression of mutant SOD1, is the principle driver of muscle atrophy.

Skeletal muscle mitochondrial defects have been reported in *MLC/SOD1*^*G*93*A*^ mice as well as in other mouse models of ALS (Dupuis et al., 2003, 2004; Dobrowolny et al., 2008). Previous studies have suggested that mitochondrial defects may lead to motor neuron degeneration in the context of ALS. This notion is supported by the study of Dupuis et al. (2009) in which they demonstrate that muscle mitochondria uncoupling leads to muscle denervation and motor neuron degeneration. Furthermore, muscle mitochondria uncoupling exacerbates disease progression and survival in a mouse model of ALS (Dupuis et al., 2009).

### Myogenesis in ALS

Altered expression of the myogenic program has previously been reported in a mouse model of ALS. Widespread differences in the transcript and protein levels of Pax7 and myogenic regulatory factors were reported at disease onset (Manzano et al., 2011). The up-regulation of these proteins may reflect an increase in satellite cell activation following myofiber degeneration that may occur at time of disease onset. The levels of myogenic regulatory factors were not differentially expressed in early and late pre-symptomatic ALS mice compared to controls. This result suggests that the aberrant expression of the myogenic program is unlikely to be a triggering event leading to skeletal muscle defects in ALS model mice. In a separate study, however, primary myoblasts isolated from ALS patients and induced to differentiate into myotubes showed decreased expression of MHC, and displayed fusion defects (Pradat et al., 2011). These results are reminiscent of what was observed in SMA and SBMA cell culture studies (Arnold et al., 2004; Shafey et al., 2005; Malena et al., 2013).

### Mechanisms of atrophy in ALS

The most overt symptom in ALS patients is muscle weakness, which ultimately leads to death. Understanding mechanisms of muscle atrophy in ALS, i.e., whether muscle atrophy in ALS is solely due to denervation or whether it is intrinsic, may offer potential therapeutic avenues able to alleviate atrophy-induced weakness. Léger et al. (2006) studied signaling pathways contributing to skeletal muscle atrophy using ALS human samples as well as ALS *SOD1*^*G*93*A*^ model mice. A dramatic increase in the expression of the E3 ubiquitin ligase atrogin 1 was detected in both mouse and human ALS samples. No changes were observed in the expression of MuRF1 (Table 1) (Léger et al., 2006). Changes upstream of atrogenes have also been reported. IGF-1 levels were decreased and the expression of activated Akt was down-regulated suggesting that muscle atrophy was associated in part to intrinsic defects not associated with myogenin-induced atrophy during denervation (Léger et al., 2006; Lunetta et al., 2012). Further evidence demonstrating that muscle atrophy is not entirely neurogenic in nature comes from studies with *MLC/SOD1*^*G*93*A*^ mouse models of ALS in which restricted mutated SOD1 expression to skeletal muscle leads to atrophy (Dobrowolny et al., 2008). Skeletal muscle atrophy in these mice is initiated by the Akt pathway which suppresses protein synthesis and induces FoxO3 mediated expression of atrogenes (e.g., atrogin1 and MuRF1) (Dobrowolny et al., 2011). Furthermore, transcript levels of genes in the autophagic pathway such as LC3, Bnip3, and CathepsinL (Table 1) are up-regulated in muscles from *MLC/SOD1*^*G*93*A*^ animals and may contribute to decreased myofiber size while caspases are likely end stage contributors to atrophy in these mice (Dobrowolny et al., 2008, 2011). These pathways altered in ALS conditional mice differ from the myogenin-induced atrophy observed following denervation. This supports the idea that intrinsic atrophy mechanisms are contributing to decreased myofiber size in ALS and that therapeutic approaches to reverse atrophy and increase myofiber size may prove beneficial in this disease. Based on these studies, it is difficult to overlook the importance of skeletal muscle as a contributor to the pathogenesis of ALS.

### Skeletal muscle IGF-1 as a therapeutic approach in ALS

The muscle-specific overexpression of IGF-1 has led to remarkable improvement in ALS model mice. Using the muscle *MLC* promoter, Dobrowolny et al. (2005) overexpressed IGF-1 onto the *SOD1*^*G*93*A*^ mouse model of ALS (*SOD1*^*G*93*A*^*/mIgf-1*). This delayed disease onset and increased survival. Muscle atrophy was attenuated and satelitte cell activation was enhanced in *SOD1*^*G*93*A*^*/mIgf-1* mice (Dobrowolny et al., 2005). Importantly, IGF-1 overexpression in muscle also preserved neuromuscular junction integrity and protected motor neurons from degeneration.

It has also been reported that viral delivery of IGF-1 to skeletal muscles leads to increased survival and protected motor neurons in ALS model mice (Kaspar et al., 2003). Interestingly, muscle viral delivery of IGF-1 in ALS model mice combined with exercise has a remarkable synergistic effect leading to an increase in survival beyond what was obseved following gene therarpy or exercise alone (Kaspar et al., 2005). It remains to been seen whether the adminstration of IGF-1 gene therapy combined with treatment of exercise mimetics such as AICAR and GW501516, can yeild similar results since exercise is likely not a feasable longterm option in severe ALS patients. Despite significant improvements in ALS model mice achieved by genetic or viral overexpression of IGF-1, to date efficacy studies of recombiant IGF-1 have yeilded positvie results in two human trials while a third trial reported no improvements in muscle strength. However, no trial has demonstated any imporovments regarding patient survival (Beauverd et al., 2012).

## Spinal and bulbar muscular atrophy (SBMA)

### Disease characteristics

Spinal and bulbar muscular atrophy (SBMA), also known as Kennedy's disease, is an adult onset neuromuscular disease characterized by the degeneration and loss of lower motor neurons leading to muscle wasting. The initial phase of the disease presents as muscle cramping, hand tremors and fatigue, with muscle weakness considered a late manifestation of disease (Sambataro and Pennuto, 2012). In addition to muscle weakness, SBMA patients display other symptoms such as fasciculations that are especially prevalent in the face, neck and tongue. The disease affects an estimated 1–2 per 100,000 people (Katsuno et al., 2012).

At the molecular level, SBMA is caused by the expansion of a polyglutamine (polyQ)-encoding CAG trinucleotide repeat in the first exon of the gene coding for the androgen receptor (AR). These repeats are toxic and lead to motor neuron death causing respiratory weakness in SBMA patients (Bricceno et al., 2012a). Motor neurons express high levels of the AR relative to other neuronal populations, and the loss of AR function attributed to the expanded polyQ tract is believed to contribute to SBMA pathogenesis. However, the predominant disease mechanism involves a gain-of-toxic function that accompanies the expanded polyQ tract AR in motor neurons (Katsuno et al., 2012).

A correlation exists between the number of repeats and the age at onset of muscle weakness (Igarashi et al., 1992). Full disease manifestations are observed in men only while heterozygous females are mostly asymptomatic and women homozygous for the mutation are rare and show only mild symptoms (Schmidt et al., 2002). The AR gene is located on the proximal arm of chromosome Xq11-12 (Katsuno et al., 2012). The AR is a nuclear receptor and is part of the steroid/thyroid hormone receptor family. Upon binding to its natural ligands testosterone and dihydrotestosterone, the AR is translocated to the nucleus where it binds DNA as well as transcriptional co-regulators to control the expression of a subset of genes. However, pathological CAG triplet repeats in the AR lead to the nuclear accumulation of the receptor and this disrupts its transactivation domain's ability to interact with transcriptional co-activators. The AR is required for androgen dependent changes including proper male pubertal sexual development. SBMA patients demonstrate subtle androgen sensitivity highlighted by gynecomastia, fertility complications and atrophy of the gonads (Sambataro and Pennuto, 2012). Castration of SBMA model mice prevents any phenotype while treating female animals with testosterone exacerbates the phenotype (Katsuno et al., 2002). These results demonstrate that disease manifestations are androgen-dependent and this explains the gender bias observed in SBMA patients.

### Muscle defects in SBMA patients

Although SBMA is believed to be primarily a motor neuron disease, increasing evidence suggests that myogenic defects contribute significantly to the disease pathogenesis. Studies performed on SBMA patients report very high levels of serum creatine kinase (CK), which are often 10 times higher than normal (Lee et al., 2005). It has been demonstrated that CK levels are higher in SBMA than any other motor neuron or myopathic disease and the increase can be detected prior to the onset of SBMA clinical symptoms (Chahin and Sorenson, 2009). Elevated serum CK levels are indicative of muscle damage and often seen in muscular dystrophy patients.

Histological studies performed on SBMA patients' muscle biopsies have revealed the presence of both neurogenic and primary myogenic defects. With muscle samples from SBMA patients, Sorarù et al. (2008), demonstrate the presence of fiber type grouping, atrophic fibers and angulated fibers, which are defects observed following chronic denervation. Necrotic myofibers as well as myofibers with centrally located nuclei, which are indicative of regenerating fibers, are also observed in SBMA muscle biopsies and are associated with primary myogenic changes (Sorarù et al., 2008; Chahin and Sorenson, 2009).

Studies using primary myoblasts isolated from SBMA patients have demonstrated that these cells proliferate and differentiate normally (Malena et al., 2013). Furthermore, primary SBMA myoblasts express myogenic regulatory factors such as MyoD and myogenin at levels comparable to controls. These results are androgen-dependent and suggest that early myogenesis is not affected in SBMA. However, defects such as cytoskeletal perturbations as well as aberrant myotube fusion were observed in primary myoblasts isolated from SBMA patients.

### Muscle defects in mouse models of SBMA

The use of mouse models has significantly contributed to our understanding of the myogenic defects in the SBMA phenotype. Yu et al. (2006) used gene targeting to generate a mouse model in which they converted the mouse AR sequence to the human sequence and in the process introduced 113 CAG repeats to recapitulate the human phenotype. These knock-in mice, designated *AR113Q*, are smaller, weaker compared to control counterparts, and die at 2–4 months of age. Histologically, muscles from *AR113Q* mice have atrophic and angulated fibers. At the molecular level, myogenin and acetylcholine receptor expression were up-regulated in *AR113Q* mice while MyoD expression was unchanged compared to controls. These morphological and gene expression changes are reflective of those observed following denervation (Moresi et al., 2010). Furthermore, myogenin was not mis-regulated in primary SBMA myoblasts supporting the idea that myogenin-dependent atrophy is present in *AR113Q* mice (Malena et al., 2013). However, the skeletal muscle pathology in *AR113Q* animals was thought to be cell-autonomous because it was detected prior to overt polyQ tract-induced pathology in the spinal cord. These results raise the possibility that the AR toxicity occurs in muscle prior to motor neurons, and may be the initiating factor leading to denervation-induced atrophy.

Other histological changes such as myofibers with centrally located nuclei present in *AR113Q* mice are more reflective of intrinsic myopathic defects. The expression of genes important for muscle function, such as the muscle chloride channel 1 (CLCN-1) and the muscle-specific sodium channel SCN4A, were decreased in *AR113Q* animals. The levels of CLCN-1 and SCN4A have previously been shown to decrease following experimental denervation (Kallen et al., 1993; Klocke et al., 1994). However, subsequent analyses in *AR113Q* animals demonstrated that the mis-regulation of CLCN-1 and SCN4A was not due to denervation (Yu et al., 2006). Furthermore, the down-regulation of CLCN-1 is attributed to the mis-splicing of the gene, which is reminiscent of what is observed in the muscle disease myotonic dystrophy (Yu et al., 2009). Therefore, SBMA model mice show muscle defects that are both intrinsic and motor neuron dependent. The denervation-like defects in *AR113Q* skeletal muscles can either be associated with intrinsic muscle toxicity-induced denervation, or to a secondary consequence linked to muscle denervation since the innervation status of skeletal muscles was not directly assessed.

Perhaps more compelling evidence supporting muscle contributions to SBMA pathogenesis comes from a mouse model in which a wild type AR was overexpressed in skeletal muscle and recapitulated the phenotypic features observed in other mouse models of SBMA (Monks et al., 2007). In this model, the AR overexpression in skeletal muscle was achieved using the *HSA* promoter (*AR-HSA*). *AR-HSA* animals showed muscle pathology and molecular changes such as increased levels of myogenin and AChR that are reminiscent of those observed following denervation. Interestingly, *AR-HSA* transgenic mice did not display motor neuron cell body loss but rather exhibited axonopathy. Again, these data suggest that the AR muscle toxicity may lead to motor neuron axon loss that in turn leads to denervation-induced pathology in skeletal muscles. Moreover, this result indicates that motor neuron defects occur distally first and eventually lead to cell body loss, a dying back phenomena, and may therefore explain the phenotype observed in the *AR113Q* mouse model of SBMA. The authors demonstrate that the toxicity to muscle occurred upon the activation of the AR-HSA by its ligand rather than the overexpression of the AR-HSA *per se*. However, it is difficult to conclude that the results observed from the *AR-HSA* mouse model can be directly generalized to SBMA, especially considering that the molecular pathogenesis of the *AR-HSA* model, which lacks the expanded polyQ tract, is very different.

### Skeletal muscle as a therapeutic target in SBMA

It has long been known that AR translocation to the nucleus is ligand-mediated and that this process is intensified in men given that the AR ligand is testosterone. Modifying testosterone levels in SBMA model mice is a ligand-targeted therapy that has proven very successful. In a mouse model of SBMA in which 97 CAG repeats were introduced into the *AR* gene (*AR97Q*), reduction of testosterone by way of castration prevented the appearance of the phenotype in male mice (Katsuno et al., 2002). In *AR97Q* female animals, administration of testosterone induced aberrant motor phenotypes and neuropathology. Treatment of *AR-HSA* female mice with testosterone provoked denervation-like symptoms similar to *AR-HSA* males. However, upon testosterone treatment cessation, *AR-HSA* females recovered normal motor function and expressed muscle genes at usual levels (Johansen et al., 2009). In agreement with these findings, survival and muscle pathology of *AR-HSA* mice is significantly ameliorated by prenatal treatment with flutamide, an AR antagonist. These data reveal that the AR expression restricted to muscle alone can lead to SBMA symptoms in mice and that these defects can be improved by blocking androgen binding. Thus, the SBMA phenotype strongly correlates with androgen expression and skeletal muscle may be an important target for SBMA treatment.

Although the use of anti-androgens is effective in treating mouse models of SBMA, it may prove to have unwanted side effects in humans. In an attempt to better understand how the AR is endogenously regulated, Palazzolo et al. (2007) uncovered a role for Akt in regulating AR function. Phosphorylation of the AR by Akt represses the AR activity through a ligand-dependent manner, thus preventing androgen from activating the AR. Modulation of Akt in cell culture models of SBMA rescued the polyQ associated cell toxicity indicating that manipulating the Akt pathway might provide phenotypic improvements in SBMA model mice (Palazzolo et al., 2007). Indeed, skeletal muscle overexpression of IGF-1, a protein that acts upstream of Akt, delayed disease onset and extended survival in SBMA model mice (Palazzolo et al., 2009). IGF-1 activates Akt and thus increases AR phosphorylation, which in turn reduces AR nuclear accumulation in muscles from SBMA model mice. A striking observation was that overexpression of IGF-1 in skeletal muscle improved motor behavior and reduced motor neuron cell loss in SBMA model mice. IGF-1 overexpression in skeletal muscle ameliorated both myofiber and motor neuron health by reducing AR nuclear aggregates. IGF-1 has previously been shown to benefit motor neurons by promoting sprouting and axonal growth, therefore IGF-1 may improve motor neuron health via its trophic properties in addition to its effects on mutant AR (Caroni and Grandes, 1990). Furthermore, overexpression of IGF-1 in skeletal muscle of wild type mice leads to gross muscle hypertrophy (Musaro et al., 2001). Therefore, IGF-1 might ameliorate muscle pathology independently of AR even though gene array data suggest that the IGF-1 signaling pathway is largely unaffected in skeletal muscle of multiple mouse models of SBMA (Mo et al., 2010).

Taken together, muscle defects are evident in SBMA and for the most part, were believed to be a secondary response to muscle denervation. However, a growing body of evidence supports the notion that muscle is the primary target of polyQ-AR toxicity and that muscle defects lead to motor neuron abnormalities. Results from various mouse models and muscle-specific targeted therapies support this idea.

## Conclusion

Although considered motor neuron diseases, the use of conditional mouse models as well cell culture systems have highlighted the contributions of intrinsic skeletal muscle defects in SMA, ALS and SBMA (Table 2). The nature of the skeletal muscle defects differs between these three diseases. For instance, the most notable muscle defects in SMA are associated primarily with impaired development but defects neurogenic in origin are also observed. While in ALS, muscle defects are due to intrinsic and neurogenic factors, and appear to be present in mature myofibers rather than in developing muscle. In SBMA, cell-autonomous muscle degeneration is an overt contributor to the phenotype as are denervation-induced defects. However, results from ALS and SBMA mouse models suggest that in these diseases, skeletal muscle defects can initiate motor neuron defects. Such evidence to support this notion in SMA is currently lacking, indeed the complete absence of Smn in skeletal muscle was not associated with motor neuron defects (Cifuentes-Diaz et al., 2001). The use of appropriate conditional animals models will serve to explore this possibility further.

**Table 2:** **Summary of defects observed in muscle in motor neuron diseases**.

		**SMA**	**ALS**	**SBMA**
Muscle physiology	Muscle atrophy	✓	✓	✓
	Reduced strength	✓	✓	✓
	Mitochondrial dysfunction		✓ ^*^	
	Atrogene expression	✓	✓ ^*^	
	Sarcomere defects	✓ ^*^		
Development	Reduced Proliferation	✓ ^*^		
	Fusion defects	✓ ^*^	✓ ^*^	✓ ^*^
	Aberrant expression of proteins important for muscle function	✓		✓
	Change in satellite cell number	✓ ^*^		
	Impaired postnatal growth	✓ ^*^		
Cell death	Increase in satellite cell apoptosis	✓ ^*^		
	Increase in cell death pathway expression	✓ ^*^		
	Myofiber degeneration			✓ ^*^

* Denotes defects that have been shown to be muscle intrinsic.

A common therapeutic approach used in all three diseases involves the muscle-specific overexpression of IGF-1. This approach has led to remarkable improvements in mouse models of ALS and SBMA, and to a lesser degree in SMA. The reason for this may be associated to the type of muscle defects in these diseases. The IGF-1 therapeutic approach may prove to be more beneficial in the context of mature muscle that is diseased, and may not be as efficient in SMA where muscle development is impaired.

It has always been assumed that muscle defects in SMA, ALS, and SBMA were a secondary consequence of motor neuron pathology. However, the studies described in this review shed light onto the importance of intrinsic muscle defects as a primary contributor to the pathogenesis of these diseases. As such, more research should be focused on treating intrinsic skeletal muscle defects. Indeed, regardless of whether muscle is a primary or secondary contributor to the pathogenesis of these diseases, muscle defects are present and therefore muscle is still an important therapeutic target to consider.

### Conflict of interest statement

The authors declare that the research was conducted in the absence of any commercial or financial relationships that could be construed as a potential conflict of interest.
